# Glial lactate metabolism and transport in Alzheimer’s disease

**DOI:** 10.3389/fnagi.2026.1731089

**Published:** 2026-03-18

**Authors:** Yaxin Wang, Boxiang Feng, Ruiwei Hong, Dan Qiu, Jinfeng Zhao, Li Zhao

**Affiliations:** 1 School of Physical Education, Shanxi University, Taiyuan, China; 2 Faculty of Health and Environmental Sciences, Auckland University of Technology, Auckland, New Zealand; 3 Baotou Teachers’ College, Inner Mongolia University of Science and Technology, Baotou, China; 4 Key Laboratory of Physical Fitness and Exercise, Ministry of Education, Beijing Sport University, Beijing, China

**Keywords:** aerobic glycolysis, Alzheimer’s disease, glial cells, lactate metabolism, lactate shuttle

## Abstract

Emerging evidence suggests that lactate, once considered merely a metabolic byproduct, plays vital roles in brain energy metabolism, signaling, and neuroprotection. In Alzheimer’s disease (AD), increasing research has implicated disruptions in glial lactate metabolism and transport as key contributors to neurodegenerative progression. This review synthesizes recent findings on the dynamic metabolic profiles of astrocytes, oligodendrocytes, and microglia, with emphasis on their stage-specific glycolytic activities and their roles in neuronal energy support. We detail how these cellular metabolic behaviors and the intercellular lactate shuttle systems—mediated by monocarboxylate transporters (MCTs) and gap junctions—are altered in AD pathology. We highlight how these changes lead to a state of neuronal energetic crisis and, paradoxically, contribute to neuroinflammation. A clearer understanding of these complex glial lactate dynamics offers a promising perspective for novel AD biomarkers and therapeutic strategies.

## 1 Introduction

Recent research has increasingly positioned Alzheimer’s disease (AD) as a metabolic disorder, with brain energy deficiency and metabolic changes identified as key causative factors (Goyal et al., 2014). A large-scale proteomic study further supports this view, revealing a protein network module associated with glucose metabolism as one of the most significant links to AD pathology and cognitive impairment (Johnson et al., 2020). A core component of brain metabolism, aerobic glycolysis, is not only crucial for energy production but also supports vital processes like biosynthesis and neuroprotection (Lunt and Vander Heiden, 2011; Goyal et al., 2014). The reduction in glycolytic flux strongly correlates with dementia severity, and its spatial distribution mirrors the deposition of amyloid β-protein (Aβ) and aberrant hyperphosphorylated tau (Vlassenko et al., 2010, 2018).

Lactate, the final product of aerobic glycolysis, was once considered merely a metabolic byproduct (Magistretti and Allaman, 2018; Brooks et al., 2022). However, its role has been reevaluated, and it is now recognized as a dynamic signaling molecule with broad influence across cellular processes and diseases. For instance, lactate can modulate immune responses by suppressing inflammatory cytokines, and within the central nervous system (CNS), it plays a multifaceted role in maintaining neural homeostasis. Its functions include regulating neuronal excitability (Cauli et al., 2023; Yao et al., 2023), promoting hippocampal neurogenesis (Nicola and Okun, 2021), and supporting synaptic function and memory formation (Rouach et al., 2008; Newman et al., 2011; Yang et al., 2014). Disrupted lactate metabolism, evidenced by altered concentrations in cerebrospinal fluid and brain tissue, is a recognized feature of AD (Lu et al., 2015; Liguori et al., 2016). Therefore, targeting lactate metabolism presents a promising and novel therapeutic strategy for treating AD.

## 2 Lactate production—aerobic glycolysis

Glycolysis serves as a critical metabolic pathway for various cell types in the brain (Cunnane et al., 2020; Wu et al., 2025), with each cell type utilizing it for distinct, specialized purposes. Among these, astrocytes are recognized as a primary player in brain glycolysis, characterized by a disproportionately high glucose uptake relative to their own energy demands (Dienel and Rothman, 2020). This high glycolytic flux is essential for supporting energy-intensive processes like the glutamate-glutamine cycle and, crucially, for maintaining the extracellular lactate pool, which acts as a vital energy substrate for neurons (Bonvento and Bolaños, 2021). Similarly, oligodendrocytes utilize glycolysis to provide energy for their own development and myelin formation, while also supplying essential substrates to adjacent neurons (Rinholm et al., 2011). Furthermore, after activation through metabolic reprogramming—a shift from oxidative phosphorylation to aerobic glycolysis—microglia primarily use glycolysis to meet the high energy requirements for their own functional remodeling and the synthesis of inflammatory cytokines (Benarroch, 2022). These distinct glycolytic activities across different cell types collectively contribute to the dynamic regulation of brain energy metabolism, particularly in the production and distribution of lactate, which is essential for intercellular communication.

### 2.1 Astrocyte

**FIGURE 1:**
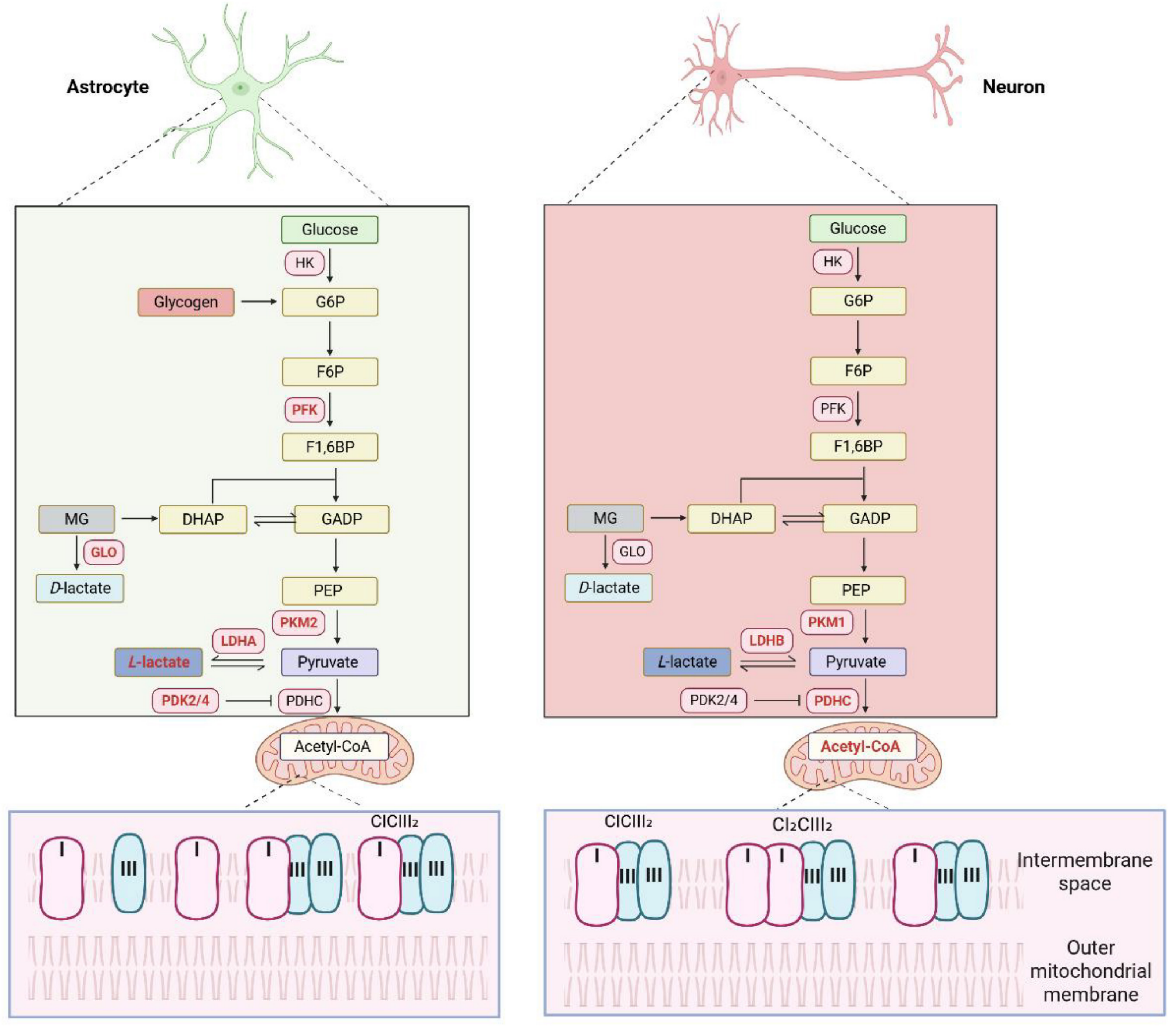
Distinct metabolic characteristics of neurons and astrocytes. Neurons do not store glycogen, whereas astrocytes are the sole glycogen-storing cells in the brain. Glycolytic enzyme activity is higher in astrocytes than in neurons. Additionally, astrocytes possess a superior capacity to detoxify glycolytic by-products and exhibit structurally distinct mitochondrial supercomplexes that contribute to their relatively lower oxidative phosphorylation efficiency. These metabolic features limit the glycolytic capacity of neurons but promote active glycolysis in astrocytes.

Astrocytes are the most abundant glial cells in the CNS and are considered the principal regulators of brain energy metabolism, primarily driven by their high rate of aerobic glycolysis (Nagase et al., 2014). This metabolic property is underpinned by several key features (Figure 1). Although the overall glycogen content in the CNS is lower than in peripheral tissues, it constitutes the brain’s largest energy reserve at the cellular level, found almost exclusively within astrocytes (Dringen et al., 1993; Gruetter, 2003; Bélanger et al., 2011; Oe et al., 2016). While neurons express enzymes for glycogen synthesis, proteasome-dependent mechanisms keep neuronal glycogen synthase in an inactive state, preventing glycogen storage (Magistretti and Allaman, 2007; Vilchez et al., 2007). In contrast, astrocyte glycogen levels are remarkably high, exceeding intracellular glucose by 20–100-fold (Dienel and Rothman, 2020). Model calculations reveal that astrocytic glycogen metabolism is primarily coupled to neuronal function by fueling the glycolytic pumping of Na^+^/K^+^ and preserving glucose for neuronal oxidation (Rothman and Dienel, 2019). The metabolic divergence between astrocytes and neurons is further defined by differences in key enzyme activities (Magistretti and Allaman, 2015). The fate of pyruvate—whether it enters the TCA cycle or is converted to lactate—is largely determined by the activity of the pyruvate dehydrogenase complex (PDHC). PDHC activity is tightly regulated by the reversible phosphorylation of its α subunit, PDHα (Patel and Korotchkina, 2006), a reaction catalyzed by a family of four pyruvate dehydrogenase kinases (PDK1-4) (Bowker-Kinley et al., 1998). Astrocytes maintain a lower PDHC activity than neurons due to the high expression of PDK2/4, which keeps PDHα in a phosphorylated and inactive state, thereby diverting pyruvate toward lactate generation (Halim et al., 2010; Zhang Y. et al., 2014). Additionally, astrocytes exhibit low activity of the E3 ubiquitin ligase APC/C-Cdh1, leading to the high expression of Fructose 2,6-bisphosphatase 3 (PFKFB3), a crucial positive regulator of glycolysis (Almeida et al., 2004; Liu et al., 2024). This contrasts sharply with neurons, where PFKFB3 is constantly degraded (Herrero-Mendez et al., 2009). Furthermore, distinct isoforms of key glycolytic enzymes are expressed: astrocytes predominantly express Pyruvate kinase M2 (PKM2) 2 and lactate dehydrogenase A (LDHA), while neurons express PKM1 and lactate dehydrogenase B (LDHB) (Bittar et al., 1996; Ward and Thompson, 2012; Zhang X. et al., 2024). Astrocytes also possess a more robust defense system against the glycolytic byproduct methylglyoxal (MG), with higher expression of the glyoxalase system and a greater capacity for glutathione and NADPH production, enhancing their resistance to oxidative stress (Thornalley, 2008; Toriumi et al., 2022). Finally, the mitochondrial respiratory chain in astrocytes is characterized by a high degree of decoupling, with Complex I largely unassociated with supercomplexes, leading to poor mitochondrial respiration. This contrasts with neurons, where Complex I is mostly embedded in supercomplexes, leading to high mitochondrial respiration (Lopez-Fabuel et al., 2016). These collective metabolic characteristics underscore the crucial role of astrocytes as a glycolytic hub, supporting neuronal function and overall brain energy homeostasis.

### 2.2 Oligodendrocytes

**FIGURE 2:**
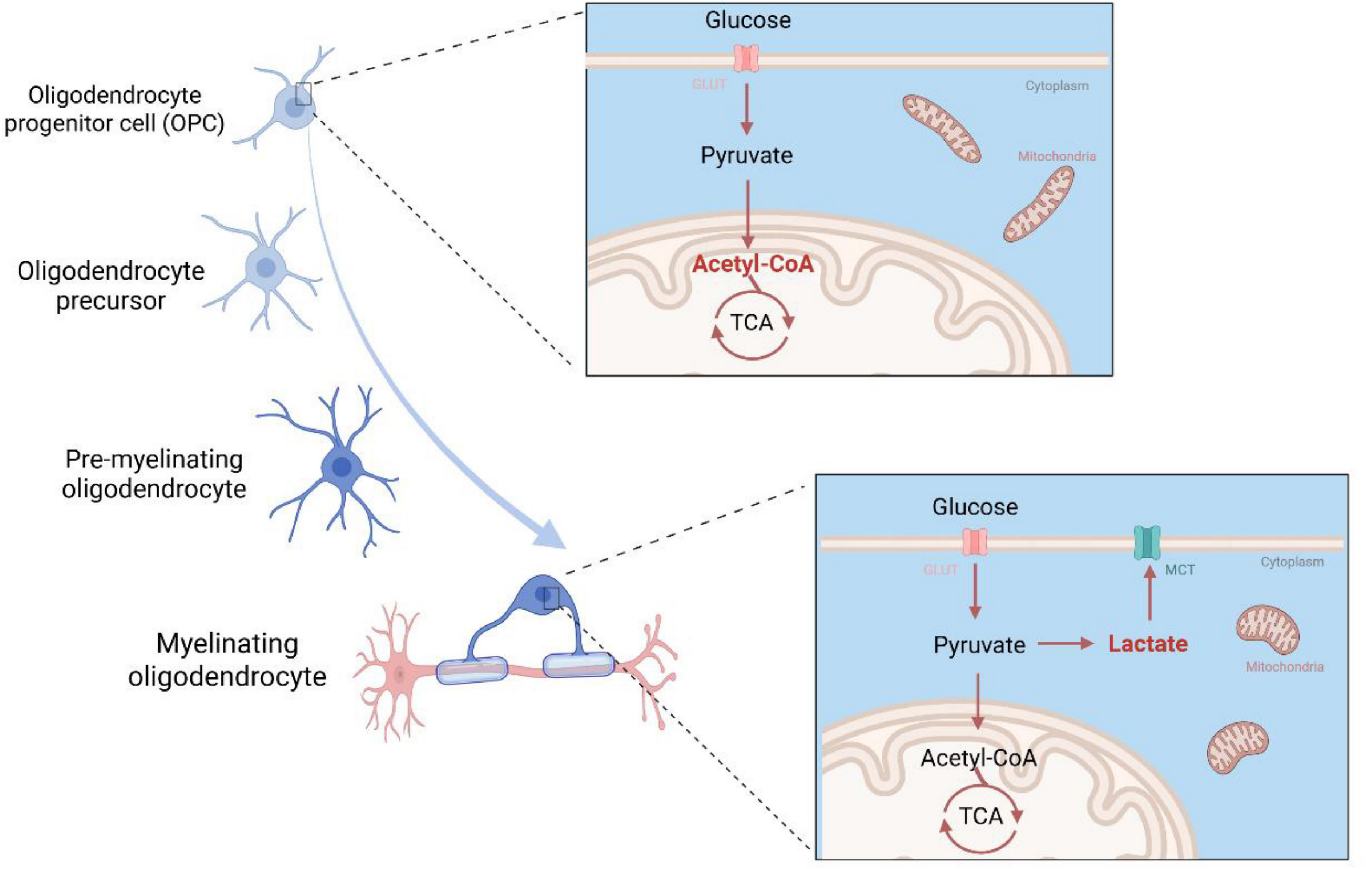
Metabolic profiles of oligodendrocytes at different differentiation stages. OPCs exhibit higher oxidative phosphorylation activity, whereas mature oligodendrocytes shift toward a more glycolysis-dominant metabolic profile.

Oligodendrocytes (OLs) are the myelinating glia of the CNS, differentiated from oligodendrocyte progenitor cells (OPCs) (Sherman and Brophy, 2005; Goldman and Kuypers, 2015). Both oligodendrocyte differentiation and the subsequent process of myelination are highly energy-dependent, requiring robust and dynamic metabolic support (Schoenfeld et al., 2010; Amaral et al., 2016). However, studies on oligodendrocyte metabolism have revealed significant discrepancies (Ziabreva et al., 2010; Fünfschilling et al., 2012), likely due to stage-specific metabolic reprogramming and inherent differences between reductionist *in vitro* and complex *in vivo* environments. Mounting evidence suggests a metabolic switch occurs during differentiation, with distinct bioenergetic profiles supporting phase-specific biological functions (Figure 2).

The metabolic properties of OPCs and OLs can be assessed by measuring the oxygen consumption rate (OCR) for oxidative phosphorylation (OXPHOS) and the extracellular acidification rate (ECAR) for glycolysis. studies on the metabolic mode of oligodendrocytes at different developmental stages may yield inconsistent results, which may be related to differences in species, cell sorting methods, or *in situ* isolation versus culture in different studies. While under optimal conditions, adult brain-derived OLs produce a greater proportion of ATP through glycolysis than OXPHOS, OCR levels increase in a linear fashion as OPCs differentiate (Narine et al., 2023). Changes in mitochondrial content, shape, and motility during oligodendrocyte differentiation are likely interdependent with metabolic transitioning. A number of studies indicate that OPCs and developing oligodendrocytes have a high density of long, tubular mitochondria, a morphology strongly correlated with high OXPHOS activity (Meyer and Rinholm, 2021). Furthermore, their mitochondria exhibit higher mobility compared to those in mature OLs, supporting the distribution of energy to growing processes and enabling the clearance of damaged organelles (Bame and Hill, 2023). These characteristics suggest a high mitochondrial metabolism and OXPHOS rate in developing oligodendrocytes before and during myelination. Conversely, mature OLs demonstrate a lower density and fragmentation of mitochondria, consistent with their reduced reliance on mitochondrial respiration. The stage-specific metabolic shifts are further reflected by the differential expression of key glycolytic enzymes. A proteome study (Fitzner et al., 2020) and a transcriptome study (Cahoy et al., 2008) showed an increase in PFKFB3 protein during oligodendrocyte development; a more complex picture emerges for other enzymes. PDH activity is significantly increased in mature OLs compared to pre-oligodendrocytes (pre-OLs) (Sajad et al., 2024). Similarly, LDHA, drives lactate production, is highly expressed in early development and during remyelination but is present at very low levels in mature oligodendrocytes (Späte et al., 2024). This metabolic reprogramming is critical for several reasons: it enables the release of lactate, which can serve as an energy substrate for ensheathed axons via monocarboxylate transporters (axon-glial metabolic coupling), limits the production of reactive oxygen species (ROS) to reduce oxidative damage, and promotes the production of carbon chain precursors essential for myelin lipid and protein biosynthesis (Brand and Hermfisse, 1997).

### 2.3 Microglia

**FIGURE 3:**
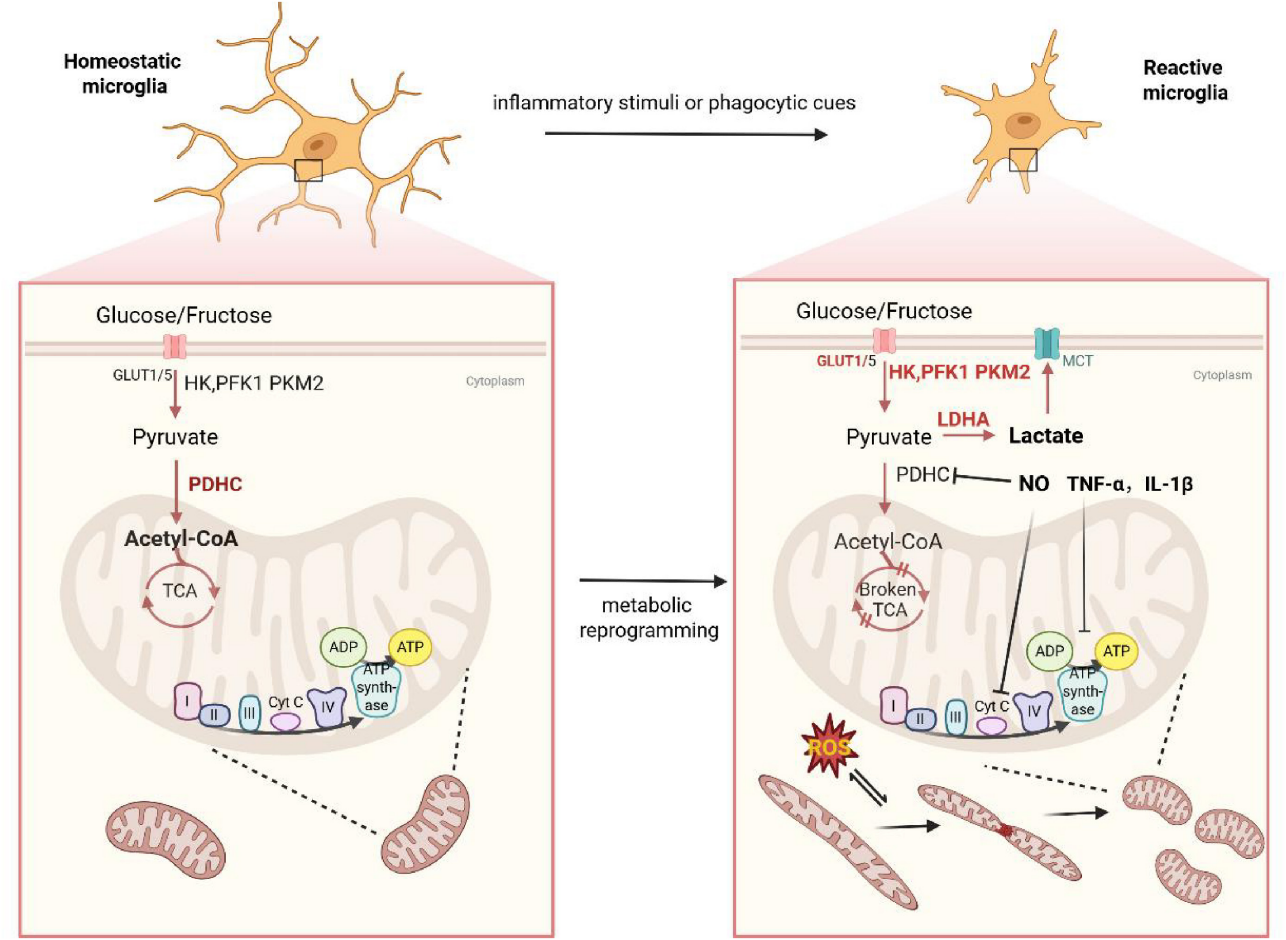
Metabolic shift toward glycolysis in activated microglia. Under homeostatic conditions or anti-inflammatory stimulation, microglia primarily rely on oxidative phosphorylation for energy production. However, upon activation by inflammatory stimuli or during phagocytic responses, microglia shift toward glycolysis. The pro-inflammatory cytokines, NO, and ROS released by activated microglia further suppress mitochondrial oxidative phosphorylation.

Microglia serve as the resident immune cells of the CNS, acting as a crucial first line of defense against harmful stimuli (Zhao et al., 2018). Their diverse functions, which include phagocytosis of apoptotic neurons and the release of neurotrophic factors, demand a constant and adaptable supply of ATP. Bioinformatic analyses have confirmed that microglia possess the genetic machinery for both glycolysis and oxidative energy metabolism, indicating significant metabolic plasticity (Zhang Y. et al., 2014). In their homeostatic, “surveilling” state, microglia rely primarily on OXPHOS for energy supply (Orihuela et al., 2016). However, upon activation by inflammatory or phagocytic cues, microglia undergo a distinct and rapid metabolic reprogramming (Figure 3). This shift toward aerobic glycolysis enables the rapid generation of ATP, which is essential for cytoskeletal remodeling, functional adaptation, and the production of pro-inflammatory cytokines that are critical for CNS injury response and repair (Aldana, 2019).

This metabolic reprogramming is intimately linked to changes in mitochondrial function and morphology. Upon activation, microglia can exhibit altered mitochondrial morphology, with some studies showing an increase in “needle-like” mitochondria (Banati et al., 2004), while others demonstrate excessive mitochondrial fission leading to fragmentation (Nair et al., 2019). This process is often driven by the activation of key fission proteins like FIS1 and Drp1, which can be triggered by inflammatory stimuli (Katoh et al., 2017). Altered mitochondrial morphology can, in turn, affect cellular function by disrupting the Krebs cycle and reducing mitochondrial calcium uptake (Zhang et al., 2013; Pereira et al., 2021).

The shift toward glycolysis is facilitated by several molecular mechanisms. Under inflammatory conditions, microglia upregulate the expression of glucose transporter 1 (GLUT1) to increase glucose uptake (Wang et al., 2019). Furthermore, activated microglia produced pro-inflammatory factors such as tumor necrosis factor (TNF)-α (Henning et al., 2023), interleukin (IL)-1β, nitric oxide (NO) (Chénais et al., 2002; Iizumi et al., 2016), and ROS (Bordt and Polster, 2014) inhibit OXPHOS. For example, NO can irreversibly inhibit the electron transport chain (Bolanos et al., 2004), while also preventing pyruvate from entering the TCA cycle by inhibiting the PDHC (Klimaszewska-Łata et al., 2015). Concurrently, the glycolytic pathway is enhanced through the increased activity and expression of key enzymes. Studies have shown an upregulation of hexokinase (HK), phosphofructokinase 1 (PFK1), and PKM2 in activated microglia, alongside a significant increase in lactate production, collectively indicating a robust enhancement of glycolysis (Gimeno-Bayón et al., 2014; Li et al., 2018). While this metabolic shift supports crucial immune functions, it may also pose a risk by affecting microglial phagocytic capacity (McIntosh et al., 2019) and promoting Aβ production (Pan et al., 2022).

## 3 Lactate transport

Lactate is a highly dynamic molecule in the brain, with its movement between cells facilitated by monocarboxylate transporters (MCTs) (Mächler et al., 2016). These proton-linked membrane proteins—primarily isoforms MCT1, MCT2, and MCT4 in the brain—enable the bidirectional transport of lactate, pyruvate, and ketone bodies across cell membranes (Hertz and Dienel, 2005). The direction and efficiency of lactate flux are dictated by concentration gradients and the distinct affinities of each MCT isoform. MCT2 possesses the highest affinity for lactate (Km: ∼0.7 mM), making it well-suited for neuronal uptake from the interstitial space. Conversely, MCT4 (Km: ∼30 mM) and MCT1 (Km: ∼3.5 mM) have lower affinities, which primarily supports lactate efflux from glial cells (Hertz and Dienel, 2005). The disruption of this specialized shuttle system can lead to a neuronal energy crisis, as vital lactate cannot be effectively delivered to support cellular function.

### 3.1 Metabolic models of lactate transport

**FIGURE 4:**
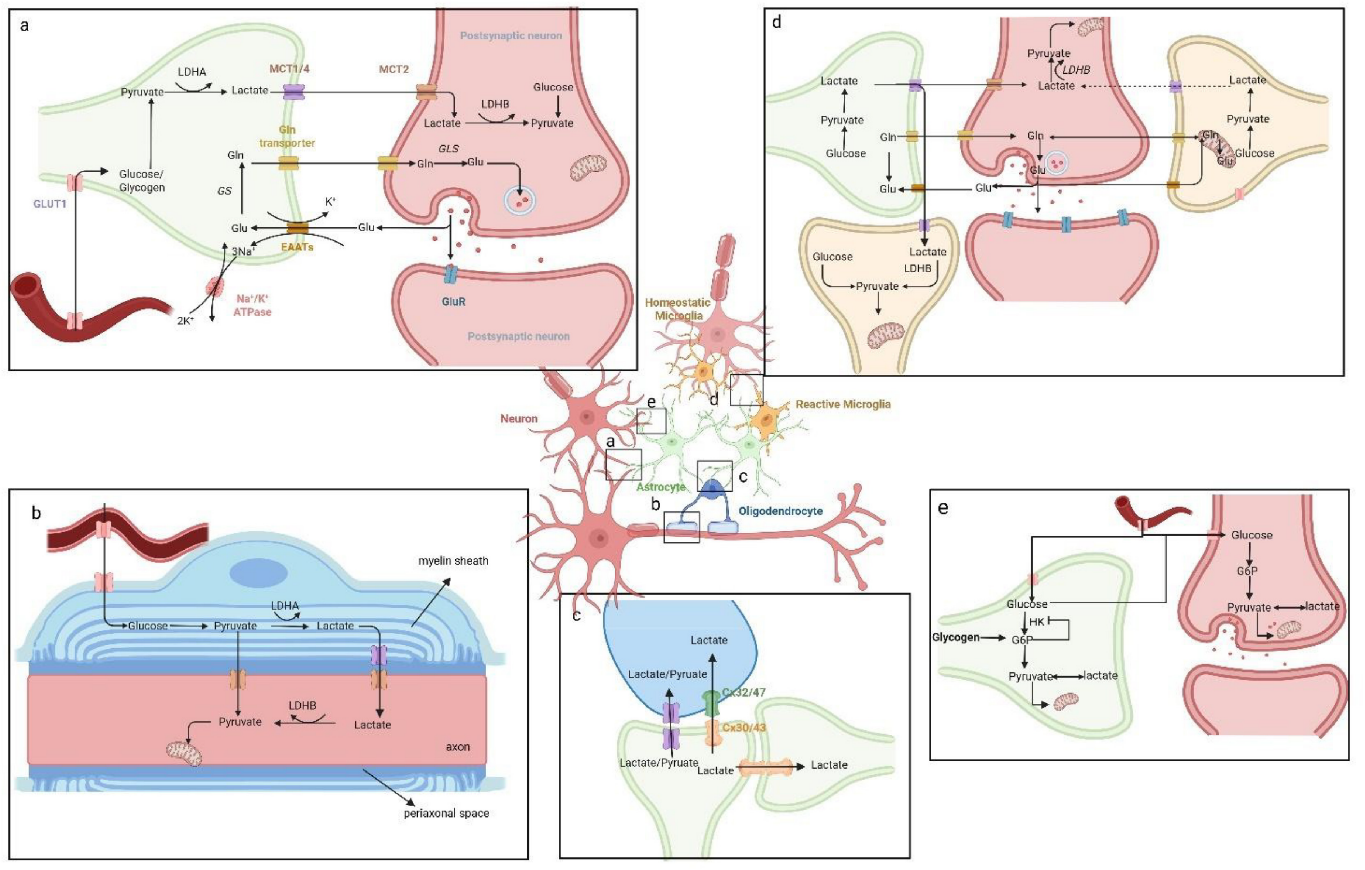
Lactate shuttling among different glial cells and neurons. **(a)** Astrocyte-neuron lactate shuttle. Lactate produced by aerobic glycolysis in astrocytes is exported into the extracellular space via monocarboxylate transporters MCT1 and MCT4. Subsequently, neurons take up lactate through MCT2, where it is converted into pyruvate by LDHB and enters the TCA cycle. In addition, the glutamate–glutamine cycle can accelerate glycolytic flux in astrocytes. **(b)** Metabolic coupling between oligodendrocytes and axons. Lactate generated by glycolysis in myelinating oligodendrocytes is directly transferred to axons through monocarboxylate transporters—MCT1 expressed in the internodal myelin sheath and MCT2 in the axoplasm—where it serves as an energy substrate, particularly under low-energy conditions. **(c)**. Lactate trafficking within astrocyte networks and between astrocytes and oligodendrocytes. Lactate diffuses through astrocytic networks via gap junction channels, enabling lactate sharing among astrocytes. Lactate shuttling between astrocytes and oligodendrocytes involves both gap junction channels and monocarboxylate transporters. **(d)** Lactate shuttle among microglia, astrocytes, and neurons. Lactate produced by astrocytes is not only taken up by neurons but also shunted to microglia, where it enters the TCA cycle to enhance oxidative phosphorylation. However, whether microglial lactate is subsequently transferred to neurons remains unclear. **(e)** Glucose sparing by glycogenolysis model. Astrocytic glycogenolysis increases intracellular G6P levels, inhibits hexokinase activity, and reduces astrocytic glucose uptake, thereby preserving extracellular glucose for neuronal oxidative metabolism.

The astrocyte-neuron lactate shuttle (ANLS) is the most extensively studied of these systems (Figure 4a; Pellerin and Magistretti, 1994). In this model, astrocytes, which are highly glycolytic, export lactate into the extracellular space via MCT4 and MCT1. Neurons, with their high capacity for oxidative metabolism, then take up this lactate via MCT2. Within the neuron, LDHB converts lactate to pyruvate, which fuels the TCA cycle to meet high-energy demands. This metabolic coupling is further reinforced by the fact that astrocytic glycolysis is enhanced when glutamate, a major excitatory neurotransmitter, is taken up (Yang et al., 2003). Astrocytes can also distribute lactate among themselves through gap junctions, creating a shared energy reservoir to meet the high metabolic demands of active synapses (Murphy-Royal et al., 2020). Beyond serving as an oxidative substrate, lactate also acts as a neuromodulator by activating lactate-sensitive receptors (e.g., HCAR1/GPR81) and regulating activity-dependent gene expression, thereby contributing to synaptic plasticity and memory formation (Lauritzen et al., 2014; Yang et al., 2014).

However, the ANLS remains a controversial hypothesis due to stoichiometric disagreements and the ability of neurons to utilize lactate. Nevertheless, a large body of experimental evidence supports the functional relevance of astrocyte-derived lactate *in vivo*. Thus, other hypotheses explaining the relationship between glucose/glycogen metabolism and neurotransmission have been proposed, including the glucose sparing by glycogenolysis (GSG) hypothesis (Figure 4e; Rothman et al., 2022). During neuronal activation, increased Na^+^ influx into astrocytes triggers rapid glycogen mobilization, leading to elevated intracellular glucose-6-phosphate levels and subsequent inhibition of hexokinase activity. This suppresses astrocytic glucose phosphorylation and reduces their reliance on blood-borne glucose, thus allowing neurons greater access to glucose provided by blood. Moreover, some of the unmetabolized intracellular glucose in astrocytes could now diffuse to neurons as supplementary fuel. Importantly, computational (DiNuzzo et al., 2010) and experimental studies (Caesar et al., 2008) indicate that glycogen metabolism induces minimal changes in intercellular lactate flux, suggesting that glycogen-derived pyruvate is largely utilized within astrocytes rather than exported as lactate.

Beyond the ANLS, other specialized shuttles support different cell types. The oligodendrocyte-neuron lactate shuttle (ONLS) is crucial due to the insulating role of myelin (Philips and Rothstein, 2017). Since myelin impedes the direct diffusion of metabolites to axons, oligodendrocytes become essential metabolic partners. MCT1, highly expressed by oligodendrocytes, facilitates lactate export to the axon, where it is taken up by axonal MCT2 (Figure 4b; Itoh, 2015). Loss of MCT1 in mature OLs causes severe late-onset axonal degeneration (Philips et al., 2021). This model of direct metabolic support, however, is a subject of ongoing debate. While some studies suggest that mature oligodendrocytes may be deficient in LDHA and thus not produce their own lactate (Späte et al., 2024), other evidence points to an indirect shuttle. In this alternative view, oligodendrocytes act as conduits, receiving lactate from astrocytes via gap junctions and then passing it on to the axons (Figure 4c; Hu and Dahl, 1999; Magnotti et al., 2011; Philippot et al., 2021).

Finally, the astrocyte-microglia lactate shuttle (AMLS) highlights the metabolic communication between these two key glial cell types. Research suggests that lactate produced by astrocytes, along with other substrates, may be preferentially directed to microglia to fuel their high-energy demands during inflammatory activation (Mason et al., 2015). Microglia are well-equipped to act as lactate consumers, with high expression of MCTs and LDHB, allowing them to rapidly oxidize extracellular lactate to fuel the TCA cycle. While the model of microglia-neuron lactate shuttle and microglia-astrocyte-neuron lactate shuttle has been proposed, it requires further experimental validation (Figure 4d; Rodríguez et al., 2013; Gimeno-Bayón et al., 2014).

### 3.2 Comparative analysis of metabolic models

Multiple lactate-related metabolic models have been proposed in the brain, and these frameworks are better interpreted as complementary mechanisms rather than mutually exclusive hypotheses. A comparative analysis can therefore be made based on their cellular focus and roles in health and disease. These models highlight the specialized roles of glial cells in supporting neuronal energy demands, intercellular communication, and metabolic homeostasis. Traditional metabolic coupling models: ANLS, AMLS, and ONLS emphasize the “shuttle” mechanism, where lactate acts as a primary energy currency and signaling molecule between glia and neurons. Whereas the GSG hypothesis primarily describes an intracellular, activity-dependent metabolic buffering mechanism in astrocytes that reallocates glucose utilization between astrocytes and neurons (Table 1).

**TABLE 1:** Comparison of ANLS/AMLS/ONLS and GSG models.

Feature	ANLS	AMLS	ONLS	GSG
Primary carbon source	Extracellular glucose (via astrocytic glycolysis); with glycogen as reserve substrate	Extracellular glucose (via astrocytic glycolysis); with glycogen as reserve substrate	Extracellular glucose (via oligodendrocytic glycolysis)	Astrocytic glycogen
Primary substrate delivered to target cells	Lactate (exported from astrocytes to neuron)	Lactate (exported from astrocytes to microglia)	Lactate (exported from oligodendrocytes to neuron)	Glucose (spared for neurons via astrocytic glycogenolysis)
Transport mechanism	MCT (MCT4/MCT1 from astrocytes, MCT2 to neurons) and gap junction between astrocytes	MCT (MCT4/MCT1 from astrocytes, MCT4/MCT1 to microglia)	MCT (MCT1 from oligodendrocytes, MCT2 to neurons) and gap junctions between astrocytes and oligodendrocytes	Minimal net intercellular lactate flux; lactate transport via MCTs may occur for intracellular–extracellular equilibrium rather than directed shuttling
Role in metabolism	Provides lactate as oxidative substrate for neuronal TCA cycle, supporting high-energy demands and glutamate-glutamine cycle	Fuels microglial oxidative metabolism and contributes to metabolic reprogramming during activation.	Supplies lactate to axons for energy, especially under low-glucose conditions, aiding myelin maintenance and lipid biosynthesis	Inhibits astrocytic glucose uptake via glycogenolysis, sparing extracellular glucose for neuronal oxidation; minimal lactate production/export
Role in signaling	Lactate acts as neuromodulator (e.g., via HCAR1/GPR81 receptors), regulates neuronal excitability, gene expression, and synaptic plasticity	Lactate modulates microglial proliferation, migration, and cytokine production (e.g., TNF-α, IL-1β), influencing neuroinflammation	No established receptor-mediated lactate signaling; signaling role remains unclear; potential HCAR1/GPR81 signaling may occur when extracellular lactate accumulates	Lactate signaling is not a primary feature; potential HCAR1/GPR81 signaling may occur when extracellular lactate accumulates
Role in neuroprotection	Supports memory-related synaptic activity and neurogenesis, and is associated with reduced neuronal energetic stress	Modulates microglial phagocytosis and immune surveillance; excessive lactate may promote inflammatory signaling and oxidative stress	Maintains axonal energy supply and myelin integrity, and is associated with preservation of white matter structure	Proposed to buffer energetic stress and support learning-related processes by preserving extracellular glucose availability during neuronal activation
Role in AD	Disruption is associated with neuronal energy deficits, synaptic dysfunction, and Aβ accumulation	Dysregulated lactate handling may contribute to altered microglial function and chronic neuroinflammation	Impaired oligodendroglial metabolic support is linked to axonal degeneration and white matter loss	Dysregulated glycogen metabolism may reduce glucose sparing, worsening neuronal vulnerability and cognitive decline

Primary substrate refers to the immediate oxidative substrate delivered to the target cell rather than its upstream metabolic origin. In ANLS, AMLS, and ONLS, lactate is derived from glucose metabolism and therefore indirectly contributes to glucose sparing.

Under physiological conditions, these models collectively ensure efficient energy distribution, neuroprotection, and synaptic maintenance, but differ in their primary cellular targets and metabolic strategies. Specifically, ANLS is primarily neuron-centric, optimizing synaptic activity and memory formation. AMLS supports immune surveillance, fueling microglial phagocytosis and cytokine production during mild stressors. ONLS is axon-specific, sustaining myelin integrity and long-distance signaling in white matter tracts. In contrast, GSG is glucose-centric rather than lactate-dependent, focusing on sparing circulating glucose for neurons during high demand, with computational models showing negligible lactate shuttling. While ANLS/AMLS/ONLS rely on glycolysis-derived lactate, GSG prioritizes glycogen mobilization, highlighting a non-glycolytic reserve mechanism.

In Alzheimer’s disease, these metabolic coupling frameworks are predicted to be differentially impaired, reflecting distinct but converging pathogenic mechanisms. Disruption of the ANLS may weaken activity-dependent metabolic support from astrocytes to neurons, thereby exacerbating neuronal energetic stress, oxidative damage, and neuroinflammation. Given that astrocyte–neuron metabolic coupling is essential for sustaining tricarboxylic acid cycle activity and neurotransmitter homeostasis, ANLS dysfunction may contribute to synaptic failure and promote amyloid-β accumulation, further accelerating disease progression (Zhang S. et al., 2024). The ONLS highlights a distinct vulnerability in white matter metabolism, whereby impaired oligodendroglial monocarboxylate support compromises axonal energy supply, contributing to axonal degeneration and white matter loss, both of which are prominent features of AD pathology (Philips and Rothstein, 2017). Although direct evidence for the AMLS in AD remains limited, accumulating studies suggest that lactate critically modulates microglial metabolic and functional states. Lactate positively regulates key microglial features, including proliferation, migration, and phagocytosis (Liu et al., 2020), and promotes reactive oxygen species production as well as pro-inflammatory cytokine expression, such as TNF-α and IL-1β, thereby participating in the modulation of synaptic plasticity and synaptic homeostasis (Andersson et al., 2005; Zhang J. et al., 2014; Monsorno et al., 2022). Moreover, excessive lactate accumulation induces histone lactylation (e.g., H4K12la), driving the formation of a glycolysis–H4K12la–PKM2 positive feedback loop, which ultimately exacerbates microglial dysfunction and neuroinflammatory responses (Pan et al., 2022). In contrast to these intercellular shuttle models, the GSG hypothesis predicts that impaired astrocytic glycogen metabolism in AD may reduce the capacity of astrocytes to buffer energetic stress and preserve extracellular glucose availability for neurons. Although direct validation of the GSG framework in AD is still lacking, growing evidence supports a critical role of astrocytic glycogen in learning, memory formation, and long-term memory consolidation (Gibbs et al., 2006; Duran et al., 2013). Moreover, disruptions of glycogen metabolism have been linked to AD, potentially due to overactivation of glycogen synthase kinase-3 (GSK-3) and subsequent inhibition of glycogen synthase, which may compromise astrocytic metabolic buffering and indirectly exacerbate neuronal vulnerability (Gibbs, 2015).

Collectively, these models suggest that lactate-related metabolic disturbances in AD arise from multi-level impairments involving astrocytic buffering capacity, intercellular substrate trafficking, immune-metabolic regulation, and axonal energy support. In the following section, we will summarize experimental evidence supporting specific alterations in lactate production and transport pathways in AD.

## 4 Lactate production and transport in AD

Clinical observations of cerebrospinal fluid (CSF) lactate levels in AD patients present an intriguing paradox, with studies reporting both decreased and significantly increased concentrations (Redjems-Bennani et al., 1998; Liguori et al., 2015, 2016; Bonomi et al., 2021). This inconsistency likely reflects the dynamic, stage-dependent metabolic changes within different glial cell populations (Table 2). However, methodological variability may also contribute to divergent findings across studies. Different analytical approaches, including enzymatic assays and gas chromatography–mass spectrometry (GC-MS), have been employed to quantify CSF lactate levels. In addition, variations in CSF collection time, storage temperature, and sample processing protocols may influence lactate measurements (Manyevitch et al., 2018). Notably, many studies did not provide detailed methodological descriptions, which may partially account for the heterogeneity of reported results.

**TABLE 2:** Lactate production and transport in AD.

Metabolic process	Biological source	Human	Animal model	Cell
Lactate production	CSF/tissue lactate	CSF lactate levels: AD patients with ApoE3 and with ApoE4↓ (Bonomi et al., 2021); control group < mild AD < moderate severe AD (Liguori et al., 2015); AD patients↑ (Liguori et al., 2016); mild cognitive impairment AD patients > dementia (Zebhauser et al., 2022); lactate levels in the posterior cingulate cortex: patients with AD↑ (Hirata et al., 2023)	Cortex and hippocampus lactate levels: 3 m APP/PS1 mice↓ (Zhang et al., 2018); Aβ_25–35_-treated rat model↓ (Lu et al., 2015); hippocampus in 6 m 3 × Tg AD mice↓ (Wang et al., 2025); dorsal hippocampus and ISF in 6 m APP/PS1 mice↑ (Yang et al., 2024); hippocampus in 12 m 5 × FAD mice↑ (Pan et al., 2022)	
	Astrocyte	sAD/fAD patients: extracellular lactate↓, ECAR↓, GLUT1 and HK1 expression↓ (Bell et al., 2025); RNA-seq profiles of post mortem AD cerebellar: HK1, IRS1 and SLC2A3 expression↓ (Saito et al., 2021)	6 m 3 × Tg AD mice: lactate↓ (Le Douce et al., 2020); reactive astrocytes isolated from the hippocampal tissues in 6 m APP/PS1 mice: lactate↑ (Yang et al., 2024)	C6 cells after Aβ_25–35_ exposure 48 h: lactate↓ (Sun et al., 2020); HiPSC-derived astrocytes after Aβ_42_ fibrils exposure 7 days: lactate↑, ECAR↑ (Zyśk et al., 2023); cultured astrocytes after Aβ_25–35_ exposure 48 h: lactate↑ (Allaman et al., 2010)
	Oligodendrocyte	RNA-seq profiles of postmortem AD brain: PFKM, PGAM1, PKM, TPI1, ENO2, and PGK1 expression↓ (Saito et al., 2021); CC1^+^ OLs in postmortem cortex sections from AD patients: HK1 expression↓ (Zhang et al., 2020)		Mature OLs after exposure to Aβ_1–42_ for 24 h: ECAR↓, lactate levels↓, HK1 expression↓ (Zhang et al., 2020)
	Microglia	RNA-seq profiles of postmortem AD brain: PFKM, PKM, TPI1, ENO2, and PGK1 expression↓, cerebellar: HK1, PFKP expression↓ (Saito et al., 2021)	Isolated adult microglia in 6 m 5XFAD mice: PKM2 expression↑, lactate levels↑, isolated microglia from 2-, 6-, or 12 m 5 × FAD mice: HIF-α, PKM2 expression↑ (Pan et al., 2022); isolated microglia from 19 to 20 m APP/PS1 mice: PFKFB3 expression↑ (McIntosh et al., 2019)	Microglia isolated from neonatal brains incubated with IFNγ (50 ng/ml) +Aβ (10 μM) for 24 h: ECAR↑, PKM2, PFKFB3, HK2 and HIF-α expression↑ (McIntosh et al., 2019)
Lactate transport		Posterior cingulate of post mortem young adult apolipoprotein E4 carriers: MCT1, MCT4 and MCT2 expression↓ (Perkins et al., 2016)	3 m APP/PS1 mice: MCT1 immunoreactivity in OLs↓, MCT4 immunoreactivity in astrocyte↓, MCT2 immunoreactivity in neuron↓ (Zhang et al., 2018); 3 m and 9 m 5 × FAD mice: Cx43 immunoreactivity↑, Cx47 immunoreactivity↓ (Angeli et al., 2020); 16 m APP/PS1 AD mice: Cx30 and Cx43 expression↑ (Cruz et al., 2010)	C6 cells after Aβ_25–35_ exposure 48 h: MCT4, GLUT1, LDHA expression↓, PC12 cells after Aβ_25–35_ exposure 48 h: MCT2, GLUT3, LDHB expression↓ (Sun et al., 2020)

“m” represent month. “↑” indicates increased/upregulated levels or expression, “↓” indicates decreased/downregulated levels or expression.

In the early, prodromal phase of AD, astrocytes are resistant to Aβ cytotoxicity, which may, in part, be related to their greater reliance on glycolytic metabolism. However, their bioenergetic capacity becomes progressively disrupted, as evidenced by impaired aerobic glycolysis and a diminished glycolytic reserve (Bell et al., 2025). This metabolic deficit compromises the astrocyte’s neuroprotective function, making it less resilient to Aβ toxicity and leading to the secretion of inflammatory factors (Garwood et al., 2011; Yan et al., 2013). However, as AD progresses, reactive astrocytes undergo a metabolic reprogramming directly triggered by Aβ oligomers (Allaman et al., 2010; Zyśk et al., 2023) and indirectly by activated microglia (Zhang et al., 2022; Yang et al., 2024). This metabolic shift leads to an accelerated, and often detrimental, overproduction of lactate. This change represents a key turning point where the astrocyte’s protective role is lost, contributing to neuroinflammation and further pathology.

Impaired oligodendrocyte glycolytic metabolism is also a significant risk factor in AD pathogenesis, contributing to early myelin degeneration and white matter loss (Nasrabady et al., 2018; Xu et al., 2022). RNA sequencing of post-mortem AD brains reveals a widespread and profound downregulation of glycolytic genes specifically within oligodendrocytes, a more extensive impairment than in any other glial cell (Saito et al., 2021). Proteomic analysis further links this deficit to the overactivation of mitochondrial dynamin-related protein 1 (Drp1), which disrupts glycolytic homeostasis. The resulting reduction in lactate production by mature oligodendrocytes contributes to axonal degeneration and white matter loss, highlighting a critical failure of the oligodendrocyte-neuron metabolic support system (Zhang et al., 2020).

Microglia, another central player in AD, undergo complex metabolic reprogramming that directly impacts their function. Acute exposure to Aβ enhances microglial aerobic glycolysis, which is crucial for their rapid activation and pro-inflammatory cytokine release (Zhao et al., 2022). However, chronic exposure to Aβ leads to a chronic tolerant phase, characterized by broad defects in both glycolysis and OXPHOS (Baik et al., 2019). This metabolic failure impairs key microglial functions, including their ability to phagocytose Aβ and maintain immunological competence.

The disruption of lactate metabolism across glial cells is compounded by a systemic failure of the lactate transport system. In AD brains, the expression of key monocarboxylate transporters is significantly altered. For instance, studies have found reduced protein levels of MCT4 in astrocytes and a significant downregulation of neuronal MCT2 (Perkins et al., 2016; Zhang et al., 2018). This impairment of the lactate shuttle system prevents efficient transfer of lactate from glia to neurons, leading to a neuronal energy crisis and a subsequent reduction in long-term memory-related proteins (Lu et al., 2019). Additionally, the reduced gap junction coupling between astrocytes further compromises their ability to distribute lactate and maintain a shared energy reservoir, which has been directly linked to impaired neuronal communication and cognitive decline (Mandal et al., 2024).

## 5 Therapeutic targeting of glial lactate metabolism in AD

Given that AD disrupts the homeostatic and neuroprotective functions of glial cells, global inhibition or activation of glycolysis risks oversimplifying their complex metabolic roles and may even worsen pathology. Accordingly, recent therapeutic strategies have shifted toward cell-type-specific metabolic restoration aimed at re-establishing glial–neuronal metabolic coupling.

### 5.1 Astrocytes

Metabolic interventions targeting astrocytes depend strongly on their activation state. In sporadic AD, astrocytic overexpression of the glycolytic enzyme HK1 markedly normalizes most metabolic abnormalities and improves cognitive performance, highlighting HK1 as a potential therapeutic target. However, HK1 overexpression fails to restore metabolism in familial AD, suggesting distinct molecular pathogenesis between AD subtypes (Bell et al., 2025). Conversely, excessive L-lactate accumulation driven by hyperglycolytic A1 astrocytes may aggravate neurotoxicity. In APP/PS1 mice, rapamycin—an mTOR inhibitor—ameliorates spatial-memory impairment and reduces Aβ plaque burden by suppressing astrocytic glycolysis-derived lactate (Yang et al., 2024).

### 5.2 Oligodendrocytes

Given the observed decline in lactate metabolism within oligodendrocytes in Alzheimer’s disease, therapeutic strategies are increasingly focusing on enhancing glycolysis in mature oligodendrocytes. For instance, suppression of Drp1 hyperactivation corrected the HK1-mediated glycolytic defect in mature OLs, reduced NLRP3 inflammasome activation, attenuated the loss of myelin and axons, and improved cognitive performance in AD mice (Zhang et al., 2020). These findings highlight the potential of targeting mitochondrial dynamics to restore energy support from oligodendrocytes to axons.

### 5.3 Microglia

Microglial phagocytic activity can be controlled by targeting their metabolism. Modulating the cellular metabolism influences phenotypic changes of microglia in both directions: activation and deactivation. Specifically, inhibition of glycolysis dramatically reduced Aβ-induced inflammatory responses of microglia. Genetic deletion or pharmacological inhibition of HK2 (lonidamine and 3-BP) (Leng et al., 2022) and PKM2 (shikonin or compound 3K) (Pan et al., 2022) promotes microglial phagocytosis, lowers the amyloid plaque burden, and attenuates cognitive impairment in male AD mice. Conversely, boosting glycolytic metabolism could restore microglial processes’ motility and increase Aβ phagocytosis. Treatment of Aβ-tolerant microglia with recombinant IFN-γ (an inflammatory cytokine and boost glycolytic metabolism) could boost the impaired glycolytic metabolism, followed by restoration of immunological functions of microglia and reverse the AD pathology *in vivo* by enhancing microglial functions (Baik et al., 2019).

### 5.4 Lactate transport

MCTs play a role in the development of AD, and targeting the repair of disrupted communication between glial cells and neurons may provide an avenue for the development of novel therapies. The combination of AMD3100 (an antagonist of CXCR4/CXCL12 signaling) and L-lactate has a beneficial effect by increasing MCT1 levels—a mechanism that explains how lactate transport into the brain improves cognitive deficits, reduces tau and APP pathologies, and promotes a shift in microglia to an anti-inflammatory M2 profile (Gavriel et al., 2022). Similarly, administration of fibroblast growth factor 21 (FGF21) can alleviate memory dysfunction, amyloid plaque pathology, and pathological tau hyperphosphorylation, and the function of FGF21 in neurodegeneration is mediated in part by the astrocyte-neuron lactate shuttle system (Sun et al., 2020). Curcumin has also been shown to ameliorate memory impairments in APP/PS1 mice, potentially by increasing brain lactate content and MCT2 expression (Lu et al., 2019).

## 6 Conclusion

The evidence presented underscores the central role of glial lactate metabolism in both brain homeostasis and the pathogenesis of AD. Astrocytes, oligodendrocytes, and microglia each exhibit unique glycolytic and lactate transport profiles that undergo dynamic, stage-specific alterations in AD. These disruptions, including the downregulation of key monocarboxylate transporters and compromised glycolysis, are closely associated with cognitive decline and neurodegeneration. Specifically, the failure of glial cells to provide neurons with lactate, either due to metabolic impairment or a breakdown of shuttle systems, precipitates a state of energetic crisis that is fundamentally linked to AD pathology.

Despite this progress, significant gaps in our understanding remain. The precise sources of neuron-available lactate and their dynamic regulation *in vivo* are still poorly defined, particularly during distinct stages of AD progression. It is also unclear whether lactate released from specific glial populations is primarily a protective mechanism or a contributor to pathology. Therefore, future research must employ advanced tools, such as cell-type-specific metabolic tracing and functional interventions, to precisely map these metabolic pathways and their roles.

Targeting lactate-related metabolic pathways, particularly through the modulation of key enzymes like PFKFB3 and HK or the regulation of MCTs, represents a promising and novel therapeutic strategy for AD. Any such approach must be carefully tailored to the specific disease stage and glial phenotype to maximize therapeutic benefits and minimize off-target effects. Ultimately, understanding and manipulating glial lactate metabolism is a critical yet underexplored frontier in AD research and drug development.
